# Delay in the Diagnosis of Patients With Head and Neck Cancer: Impact of Different Patient- and Healthcare Provider-Related Factors

**DOI:** 10.7759/cureus.68528

**Published:** 2024-09-03

**Authors:** Saleh Khurshied, Zainab Shahid, Tamoor Wazir, Imdad Ullah, Shana Sagheer, Nawal Khurshid, Altaf Hussain

**Affiliations:** 1 Otolaryngology - Head and Neck Surgery, Pakistan Institute of Medical Sciences, Islamabad, PAK; 2 Otolaryngology - Head and Neck Surgery, Akhtar Saeed Medical College, Rawalpindi, PAK; 3 Medicine and Surgery, Rawal Institute of Health Sciences, Islamabad, PAK

**Keywords:** healthcare provider, tnm stage, treatment options, delayed diagnosis, head and neck cancer (hnc)

## Abstract

Background

In our setup, head and neck cancer (HNC) is the most common, and patients frequently present at an advanced stage, which results in dismal outcomes. Delays on the part of the patient (such as resistance to seeking treatment) or the provider (such as misdiagnosis or an extended wait period for consultation) may be the cause of a late presentation. The presentation stage may vary depending on several factors, including age, gender, smoking status, job status, and education.

Objectives

The study aims to identify factors that lead to advanced-stage presentations of HNCs and to determine the delays brought on by patient- or healthcare provider-related factors and how these factors affect HNC disease staging among biopsy-proven HNC patients.

Materials and methods

Participants in the study were those who initially presented with a biopsy-verified HNC at the cancer clinic of the department of otolaryngology-head and neck surgery at Pakistan Institute of Medical Sciences (PIMS), Islamabad. Patients answered questions on their first symptom presentation, past healthcare professional visits, and intervals between visits on a Cancer Symptom Interval Measure (C-SIM) questionnaire. For every patient, clinical and demographic information was gathered. TNM staging was completed. The test of significance was applied where applicable.

Results

Age, gender, education level, and smoking status had no bearing on the presentation stage. Patients without jobs present at a statistically significant higher stage (p = 0.038). The most prevalent histological form of HNC was squamous cell carcinoma 79 (82.29%), with the oral cavity and larynx being the most common sites of the disease 30 (31.25%) and 29 (30.21%) respectively. Patients took an average of 5.28 ± 9.12 months from the onset of symptoms to their first appointment with a healthcare provider. Prior to diagnosis, the majority of patients saw three or more healthcare providers (range: 1-8). The duration from the initial visit to a healthcare provider to the initiation of treatment was 3.06 ± 5.88 months. Based on the stage at presentation, there were no discernible variations in the times to presentation (p>0.05).

Conclusion

Significant delays and high stage of presentation are caused by unemployment. The majority of the delay was caused by the patient's tardiness in seeing a medical practitioner, yet the presentation stage was not greatly impacted by this delay.

## Introduction

With over 500,000 new cases identified annually, head and neck cancer (HNC) is the fifth most common neoplasm [1]. HNC is a collection of malignant tumors with a similar biological makeup. HNCs can be classified according to their histological type (squamous cell carcinoma (SCC) being the most common type of disease) and anatomic sites of origin (nasopharynx, nasal cavity, paranasal sinuses, oropharynx, oral cavity, larynx, hypopharynx, salivary glands, and thyroid glands). With so many different possible treatment choices, the wide range of anatomical and histological heterogeneity impacts every stage of the management, from initial staging to diagnosis and prognosis [2,3].

Management of patients with advanced HNCs is still a therapeutic challenge due to the disease's variability. With a 30-35% five-year overall survival rate, the prognosis is still bleak despite numerous attempts to improve outcomes [4].

In southeast Asia, the HNC accounts for 8-10% of all cancer cases and is the most diagnosed malignancy worldwide. The primary causes of HNCs in Pakistan are a distinct demographic profile, risk factors, dietary habits, and a positive family history [5]. For HNC patients, delays in diagnosis are typical. Before seeking assistance, patients frequently ignore their symptoms for extended periods of time, and some family doctors might not be aware of the warning signs and symptoms of HNCs [6]. Patient outcomes in HNCs may be impacted by delays in diagnosing and then initiating treatment [7].

For head and neck squamous cell carcinoma (HNSCC), the time interval between diagnosis and the start of curative treatment is lengthening in the USA. This is due to the use of more advanced diagnostic and treatment methods, more care transitions, and possibly a shortage of healthcare providers (HCPs) [8]. One of the most important predictors of the fate of an HNC is the tumor stage upon presentation [9]. There is ample evidence that advanced clinical presentation can arise from a delay in the initiation of treatment [10,11]. The effect of a delayed diagnosis on outcomes related to survival and quality of life, however, is not much appreciated. Patients with poorer prognoses and more advanced-stage cancer are presumably those whose diagnoses were delayed; however, no correlation has been shown [12].

Two key phases can be distinguished in the delay prior to a head-and-neck oncologist's assessment: the patient delay and the provider delay. The interval between the onset of a new symptom and the first appointment with an HCP is known as the patient delay, which happens when people choose not to seek medical assistance right away. The time period between the patient's appointment with the HCP and their presentation at the multidisciplinary team clinic is referred to as the provider delay, which happens after the patient has established an initial visit with the first HCP for a symptom. To date, there has been little research on the overall duration of delays for patients and providers, and the reasons for these delays are mostly unclear [13].

The idea behind the study is that better treatment results for patients will arise from our ability to identify and reduce the factors that delay the identification and subsequent management of HNCs.

The study aimed to identify the many characteristics associated with HNC and investigate their relationship to the presenting stage. In order to ascertain the reasons behind the delays in HNC diagnosis and therapy, several patient- and HCP-related factors that cause delays in presentation, diagnosis, and subsequent treatment should be identified.

## Materials and methods

After receiving formal approval from the PIMS ethical research review board with reference no. F.3-1/2023(ERRB)/Chairman, this cross-sectional study was carried out in the Department of Otolaryngology-Head and Neck Surgery at the Pakistan Institute of Medical Sciences (PIMS), Islamabad, from December 1, 2023, to June 30, 2024, for a period of seven months. The study included all biopsy-proven HNC patients, regardless of age or gender, who presented to the cancer clinic. For every patient involved in the study, a signed consent form was obtained. Patients with recurrent disease and those with a prior history of HNC surgery or chemoradiotherapy were excluded. A questionnaire called the Cancer Symptom Interval Measure (C-SIM) was used to gather data [14]. The study employed a consecutive convenient sampling strategy to include all patients who visited the cancer clinic during the study period. Translating the questionnaire into the patient's native tongue, the doctor conducted the interview and completed the appropriate sections of the form. Merely the sections of the survey relevant to the respective patient were completed. The American Joint Committee on Cancer Staging (AJCC) handbook, eighth edition, was used to perform TNM tumor staging on patients [15]. On the basis of their presenting stage, the patients were split into two groups. As per the AJCC staging, the early stage included presenters in stages I and II and the advanced stage of presenters in stages III and IV. Where applicable, the mean value with standard deviation and frequency with percentages were computed for the demographics and displayed as a table. To determine the association, various factors were correlated among both groups. The Pearson chi-square or Fisher exact test was used for categorical variables, where appropriate, to compute the p-value. Mean values were compared by the independent samples t-test, and the p-value was calculated. Months were used to compute the time period. IBM SPSS Statistics for Windows, Version 27 (Released 2020; IBM Corp., Armonk, New York, United States) was used to analyze the data. A p-value < 0.05 was considered statistically significant.

## Results

During the study's duration, 96 patients in total met the inclusion requirements. Group A consisted of 21 (21.87%) patients, while Group B comprised 75 (78.13%) patients after the patients were divided into early and advanced stages, respectively. The participants' mean age was 63.07 ± 11.93. When the mean age of both groups was examined, no significant association was discovered (p = 0.552). No statistically significant difference was found among the two groups in terms of gender distribution (p = 0.732), with 76 (79.17%) male patients and 20 (20.83%) female patients. Only employment showed a statistically significant association between the two groups when factors such as education, employment status, smoking status, years smoked, and pack year were compared. This indicated that patients without jobs had a significantly higher stage of disease than those with jobs, with a p-value of 0.038. Table 1 provides specifics for every factor along with a p-value.

**Table 1:** Patients' demographic factors affecting the stage of presentation The data is presented as n (%) for the number of patients, sex, education, employment, and smoking status and as (mean ± SD) for age, years smoked, and pack years. Statistical analysis was performed using the chi-square and independent samples t-test (where applicable), with significance set at p<0.05. *Active + Ex-smokers

Factors		Group A (Early Stage)	Group B (Advanced Stage)	Overall	p- Value
Number of patients		21 (21.87)	75 (78.13)	96 (100)	-
Age (years) (mean ± SD)		61.13 ± 10.34	63.76 ± 12.22	63.07 ± 11.93	0.552
Sex	Male	18 (85.71)	58 (77.33)	76 (79.17)	0.732
	Female	3 (14.29)	17 (22.67)	20 (20.83)	
Education	Educated	7 (33.33)	21 (28)	28 (29.17)	0.490
	Uneducated	14 (66.67)	54 (72)	68 (70.83)	
Employment	Employed	13 (61.90)	26 (34.67)	39 (40.62)	0.038
	Unemployed	8 (38.10)	49 (65.33)	57 (59.38)	
Smoking status	Smoker*	15 (41.43)	51 (68)	66 (68.75)	0.807
	Non-Smoker	6 (28.57)	24 (32)	30 (31.25)	
Years smoked (mean ± SD)		25.87 ± 11.45	28.14 ± 14.61	27.78 ± 14.04	0.467
Pack year (mean ± SD)		25.32 ± 21.76	28.22 ± 26.01	27.53 ± 25.97	0.432

The features of the various HNC patients involved in the study are displayed in Table 2. SCC was the most prevalent histological type of tumor overall, accounting for 79 (82.29%) of cases. The oral cavity 30 (31.25%) and larynx 29 (30.21%) were the two most frequent tumor sites. The majority of patients had advanced stages, with 42 (43.75%) having stage IV and 33 (34.37%) having stage III. Table 2 displays the specifics of TNM staging.

**Table 2:** Characteristics of head and neck cancer The data is presented as n (%) SSC: Squamous cell carcinoma; AJCC: The American Joint Committee on Cancer Staging

Characteristics		Early Stage	Advanced Stage	Overall
Histological type of cancer	SCC	17 (80.95)	62 (82.67)	79 (82.29)
	Others	4 (19.05)	13 (17.33)	17 (17.71)
Site of cancer	Oral cavity	5 (23.81)	25 (33.33)	30 (31.25)
	Oropharynx	2 (9.52)	8 (10.67)	10 (10.42)
	Nasal cavity and/or Paranasal sinuses	1 (4.76)	3 (4)	4 (4.17)
	Hypopharynx	3 (14.29)	9 (12)	12 (12.5)
	Larynx	7 (33.33)	22 (29.33)	29 (30.21)
	Salivary glands	2 (9.52)	5 (6.67)	7 (7.29)
	Thyroid	2 (9.52)	3 (4)	5 (5.21)
Stage as per AJCC at the time of presentation	I	8 (38.09)	33 (44)	8 (8.33)
	II	13 (61.91)	42 (56)	13 (13.54)
	III	0 (0)	0 (0)	33 (34.37)
	IV	0 (0)	0 (0)	42 (43.75)
T stage at the time of presentation	T1	5 (23.81)	2 (2.67)	7 (7.29)
	T2	16 (76.19)	17 (22.67)	33 (34.37)
	T3	0 (0)	20 (26.67)	20 (20.83)
	T4	0 (0)	36 (48)	36 (37.5)
N stage at the time of presentation	N0	19 (90.48)	15 (20)	34 (35.42)
	N1	2 (9.52)	31 (41.33)	33 (34.37)
	N2	0 (0)	23 (30.67)	23 (23.96)
	N3	0 (0)	6 (8)	6 (6.25)
M stage at the time of presentation	M0	0 (0)	51 (68)	51 (53.12)
	M1	0 (0)	18 (24)	18 (18.75)
	Mx	0 (0)	8 (10.67)	8 (8.33)

When the numbers of HCP visits prior to the diagnosis of HNC were examined between the two groups, there was no statistically significant correlation between them (p = 0.106). Of the HCP visits, 39 (40.62%) had visited less than three, and 57 (59.38%) had visited three or more times. As indicated in Table 3, the mean number of months from the onset of symptoms to the first HCP visit, the first HCP visit to first imaging, the first HCP visit to biopsy, the first HCP visit to first visit to cancer clinic, and the first HCP visit to treatment initiation were, respectively, 5.28 ± 9.12, 1.07 ± 3.01, 0.64 ± 0.76, 0.25 ± 0.36, and 3.06 ± 5.88 with no statistically significant difference between the two groups showing that most of the delay was due to patient factor.

**Table 3:** Delay in the diagnostic route and patient and healthcare provider factors The data is presented as n (%) for number of HCP seen before diagnosis and (mean ± SD) for all other characteristics. Statistical analysis was performed using the Chi-square and independent samples t-test (where applicable), with significance set at p<0.05. HNC: Head and neck cancer; HCP: healthcare provider; N: number; USG: ultrasonography; MRI: magnetic resonance imaging; CT: computed tomography; SD: standard deviation; CRT: chemoradiotherapy

Characteristics		Group A (Early stage)	Group B (Advanced stage)	Overall	p-Value
Number of HCPs seen before diagnosis	<3	15 (71.43)	24 (32)	39 (40.62)	0.106
	≥3	6 (28.57)	51 (68)	57 (59.38)	
Months taken from the first onset of symptom to the first visit to the HCP (mean ± SD)		3.89 ± 12.40	5.71 ± 8.34	5.28 ± 9.12	0.568
Months taken from the first visit to HCP to the first imaging (USG, MRI, and CT scan) done (mean ± SD)		1.12 ± 2.77	1.04 ± 3.11	1.07 ± 3.01	0.998
Months taken from the first visit to HCP to biopsy (mean ± SD)		0.67 ± 0.78	0.63 ± 0.75	0.64 ± 0.76	0.953
Months taken from diagnosis to the first visit to HNC clinic (mean ± SD)		0.29 ± 0.41	0.24 ± 0.34	0.25 ± 0.36	0.874
Months taken from the first visit to HCP and start of treatment (Surgery/CRT) (mean ± SD)		2.78 ± 4.99	3.11 ± 6.44	3.06 ± 5.88	0.589

## Discussion

According to this study, 75 (78.13%) of the patients had advanced stages when they were first seen. In contrast, it was 50% in the study by Kassirian et al. [13]. The mean age of the HNC patients in our study was 63.07 ± 11.93, which was consistent with findings from previous HNC investigations [3,13,16,17]. Similar to the findings of earlier research, the majority of our patients were men [3,13,16,17]. With the exception of unemployment, we were unable to identify any other factor that was substantially contributing to the advanced stage of presentation, delaying diagnosis and treatment, as confirmed by the findings of other research studies [18-21].

The study found that patient variables were the main reason for the delay in diagnosis; the average time from the start of initial symptoms to the first visit with the HCP was 5.28 ± 9.12 months, which is in line with other studies' findings [22-25], that the time frame was between 3.7 and 4.3 months. Considering other studies, our HCP delay was 3.06 ± 5.88, which is relatively short [6,13,18,22,24]. This included diagnostic investigations, multidisciplinary meetings, and the beginning of treatment. We discovered, in line with research findings [13,24], that there was no correlation between the two groups in terms of delay. The HCP delay in Canada was reported in the literature to be 11 months, while it was roughly 3-5 months in the UK and three months in the US [9,13,26,27].

According to our study, 82.29% of the histological types were SCC, which was also the most common HNC in the literature where its prevalence was approximately 90% [28-31]. According to studies, the oral cavity and oropharynx were the most frequently observed locations of HNC in our study [13,18,29]. Similarly, the oral cavity is the most commonly involved site in our study 30 (31.25%).

Our study's strengths were a large sample size and the use of a previously approved questionnaire. We used a consecutive sampling technique. We conducted our study using a prospective design, meaning that patients who came to see us for the first time were monitored till the end.

Our study's limitations were the participation of patients from a single center. Another drawback was recall bias; however, this was reduced by employing a prospective study design and a validated questionnaire. A further constraint was that the amenities found in a tertiary care setting such as ours are not found in many hospitals; rather, they are found in a very small number of institutions, making it challenging to extrapolate the findings.

## Conclusions

With the exception of unemployment, where patients had advanced disease when presented, we discovered that the majority of characteristics had no effect on the timing of HNC diagnosis. The oral cavity and larynx were the most often affected areas, with SCC being the most prevalent HNC. Most patients showed up at a later stage. We found that the patient presented with a large delay, with the patient-related factors contributing to the longest delay. The advanced stage of the disease's manifestation did not appear to be impacted much by the delay.
